# Daily Activity Engagement Predicts More Positive Affect Especially on Days When a Person Feels Young

**DOI:** 10.1093/geroni/igab046.3274

**Published:** 2021-12-17

**Authors:** Erica O'Brien, Shevaun Neupert

**Affiliations:** 1 Pennsylvania State University, University Park, Pennsylvania, United States; 2 North Carolina State University, Raleigh, North Carolina, United States

## Abstract

Engagement in a wide array of mental, social, and physical leisure activities confers several health benefits. Indeed, theories of successful aging argue that an active lifestyle serves as an important criterion for maintaining high levels of psychological, functional, and physical well-being in old age. Findings from parallel studies also show that people who hold positive (self-)views of aging exhibit higher and maintained levels of well-being over time. Yet, whether views of aging enhances the link between activity engagement and well-being - and whether they do so on a daily basis – remains unknown. This study therefore sought to extend prior literature by examining the relationship between activity engagement, subjective age, and affective ratings within-person over several days. Old adults (N = 115; Age: Range = 60 – 90, M = 64.65, SD = 4.86) in the Mindfulness and Anticipatory Coping Every Day (MACED) study completed an 8-day daily diary. Participants reported on their positive and negative affect, the age they subjectively felt compared to their actual age, and the number and types of leisure activities in which they engaged. Results from multilevel analyses indicate that people felt more positive on days when they also engaged in more activities (total across mental, social, physical types) than usual. Moreover, the effect of activity engagement was most pronounced on days when people felt younger than usual. No effects were found for negative affect. Preliminary findings suggest that people benefit psychologically from daily leisure activities and a positive self-view of aging.

